# Comparative Clinical Study Between Modified Ureteral Orthotopic Reimplantation and Cohen Method Under Pneumovesicum in Pediatric Patients With Hydroureteronephrosis

**DOI:** 10.3389/fped.2020.00062

**Published:** 2020-03-06

**Authors:** Jiaming Chang, Qiangye Zhang, Peimin Hou, Dongming Wang, Aiwu Li, Xiaona Lv

**Affiliations:** Department of Pediatric Surgery, Qilu Hospital, Shandong University, Jinan, China

**Keywords:** primary obstructed megaureter, vesicoureteral reflux, comparative study, ureteral reimplantation, pneumovesicum, pediatric patients

## Abstract

**Purpose:** To report our initial experience with a modified ureteral orthotopic reimplantation technique under pneumovesicum and compare the outcomes vs. those obtained with the Cohen technique under pneumovesicum for the correction of primary obstructive megaureter (POM) or vesicoureteral reflux(VUR) in pediatric patients.

**Methods:** A total of 46 patients (38 POM and 8 VUR; mean age: 16.24 months) treated with modified ureteral orthotopic reimplantation (OR) and 43 patients (34 POM and 9 VUR; mean age: 22.98 months) treated with Cohen reimplantation (CR) under pneumovesicum were included. We compared the results perioperatively and during follow-up.

**Results:** The mean operative time was significantly shorter in the OR group (OR: 86.86 and 108.18 vs. CR: 95.14 and 124.29 min for unilateral and bilateral cases, respectively). The mean postoperative hospital stay (OR: 5.02 vs. CR: 5.07 days), blood loss (OR: 3.67 vs. CR: 3.84 ml), and follow-up time (OR: 23.17 vs. CR: 23.37 months) did not exhibit significant differences between the two groups. One patient converted to open surgery in the CR group, whereas there was no conversion in the OR group. Postoperative febrile urinary tract infection occurred in two cases in each group. Both infections were controlled using antibiotics. All patients in both groups showed improved hydroureteronephrosis, and all patients with VUR showed reflux resolution post-surgery.

**Conclusions:** Our modified ureteral orthotopic reimplantation technique under pneumovesicum can be safely and effectively performed, achieving a high success rate that is equivalent to that obtained through the Cohen technique under pneumovesicum. Moreover, it involves a simpler procedure and shorter operation time.

## Introduction

Primary obstructive ureter (POM) and primary vesicoureteral reflux (VUR) are congenital malformations of the upper urinary tract in children. In healthy children, the estimated prevalence of VUR is 0.4–1.8% (1), while that of POM is 0.36 in 1,000–1,500 live births (2, 3). There is considerable controversy regarding the management of VUR and progressive POM. For POM or low-grade (grades I–II) VUR, the majority of cases resolve spontaneously or persist without deterioration of renal function and appearance of symptoms (4, 5). Surgical intervention should be considered in patients with high-grade (grades IV–V) VUR (1) or those cases associated with urinary tract infection (UTI), increasing dilatation, and deteriorating renal function (2, 3). Lengthening of the intramural part of the ureter by ureteral tapering and reimplantation is an established and reliable treatment for high-grade VUR and persistent POM. However, performing classic reimplantation in the bladders of infants may be extremely challenging due to the limited bladder volume. Moreover, this procedure is linked to difficulty in endoscopically accessing the ureters in the future (6). We aimed to present our initial experience with a modified ureteral orthotopic reimplantation technique under pneumovesicum and report the clinical outcomes compared with those obtained using the Cohen technique under pneumovesicum for the correction of POM and VUR.

## Patients and Methods

### Patient Characteristics

From January 2015 to December 2018, 46 patients (10 girls and 36 boys; mean age: 16.24 months; range: 2 months−6.4 years) underwent modified ureteral orthotopic reimplantation (OR) under pneumovesicum, and 43 patients (10 girls and 33 boys; mean age: 22.98 months; range: 3 months−6.9 years) underwent Cohen reimplantation (CR) under pneumovesicum. Among them, 38 and 8 patients were diagnosed with POM and VUR, respectively, in the OR group; in the CR group, these numbers were 34 and 9 patients, respectively. In the OR group, 35 and 11 cases were unilateral and bilateral, respectively; in the CR group, these numbers were 36 and seven cases, respectively (Table 1). POM or VUR was diagnosed after confirming the absence of ureteropelvic junction obstruction and the presence of hydroureteronephrosis through magnetic resonance urography. Patients were evaluated preoperatively with at least two urinary system ultrasounds, voiding cystourethrography (VCUG) (**Figure 2A**), and magnetic resonance urography (**Figure 2B**). Surgery was performed solely under the following conditions for POM: ≥10% deterioration of split renal function; diameter of the megaureter ≥1.0 cm; and recurrent febrile UTI after routine anti-infective therapy. For patients with VUR, operative indications were as follows: high-grade reflux on VCUG (grades IV–V); recurrent febrile UTI after routine anti-infective therapy; or ≥10% deterioration in split renal function. All unilateral patients showed normal renal function in the healthy side on magnetic resonance urography. The surgical methods were identical for patients with POM and VUR. Operative methods were discussed with the guardians of the patients, and informed consent was provided prior to the surgery.

**Table 1:** Comparison of patient characteristics between modified ureteral orthotopic reimplantation (OR) and Cohen reimplantation (CR).

**Characteristic**	**OR (*n* = 46)**	**CR (*n* = 43)**	*p*-value^*^
Age, mean ± SD (months)	16.24 ± 14.55	22.98 ± 21.80	0.08
Sex, *n*(%)			
Male	36 (0.78)	33 (0.77)	0.86
Female	10 (0.22)	10 (0.23)	
Laterality, *n*(%)			
Unilateral	35 (0.77)	36 (0.83)	0.37
Bilateral	11 (0.23)	7 (0.16)	
Etiology, *n*(%)			
POM	38 (0.83)	34 (0.79)	0.76
VUR	8 (0.17)	9 (0.21)	

* *p-values calculated using the chi-squared test for categorical variables and Student's t-test for continuous variables. SD, standard deviation; POM, primary obstructive megaureter; VUR, vesicoureteral reflux*.

### Surgical Techniques

#### Pneumovesicoscopic Modified Orthotopic Reimplantation

**Figure 1:**
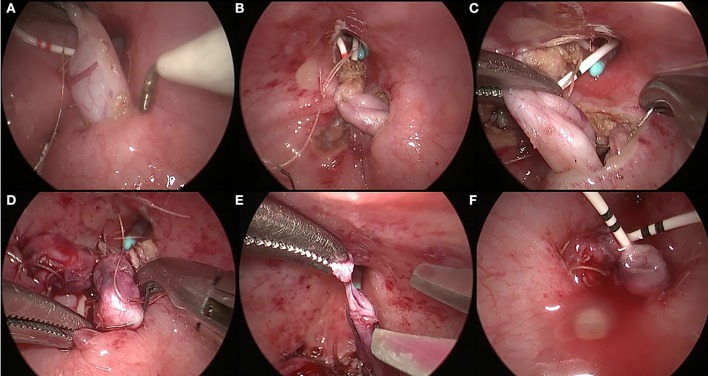
Surgical procedures of pneumovesicoscopic-modified ureteral orthotopic reimplantation. The distal ureter was progressively dissected free from the bladder wall until the dilated proximal segment **(A)**. The free segment of the ureter was dragged into the bladder for 4–5 cm and suspended on the contralateral bladder wall **(B)**. The muscle of the bladder was sutured to the ureteral seromuscular layer using 5-0 absorbable interrupted sutures **(C)**. The bladder mucosa was sutured to the ureteral seromuscular layer using 5-0 absorbable interrupted sutures **(D)**. The distal lesion segment was excised at the transitional segment of the ureter **(E)**. The proximal dilated ureter protruded into the bladder for 2–2.5 cm at the original position and became the neo-orifice with a double J stent in the ureter **(F)**.

After the induction of general anesthesia, the patient was placed in the supine position with the legs separated. The bladder was distended with saline and anchored to the abdominal wall using a stay suture under cystoscopic vision. A 5-mm trocar (camera port) was placed first into the bladder, followed by two additional 3-mm trocars inserted into the bladder on either side of the lower lateral wall. After removing the cystoscope and draining the saline in the bladder, the CO_2_ pneumovesicum was established at a pressure of 10–12 mmHg. The urethral catheter served as an occlusion of the internal urethral meatus to secure the CO_2_ pneumovesicum and an additional suction or flushing device during the subsequent operation procedure. Under the guidance of a laparoscope, a segment of a 3F ureteral stent was inserted 4–6 cm into the respective ureter and secured with a 5–0 absorbable suture for subsequent ureteral dissection. The distal ureter was progressively dissected free from the bladder wall until the dilated proximal segment using electrocautery (Figure 1A). Using a blunt grasper to apply traction to the ureteral stent, the free segment of the ureter was dragged into the bladder for 4–5 cm and suspended on the contralateral bladder wall (Figure 1B). Subsequently, the muscle of the bladder and bladder mucosa was sutured to the ureteral seromuscular layer using 5–0 absorbable interrupted sutures (Figures 1C,D). Next, the distal lesion segment was excised at the transitional segment of the ureter (Figure 1E). Finally, the proximal dilated ureter was embedded 1–1.5 cm between the bladder mucosa and the muscle of the bladder, protruding into the bladder for 2–2.5 cm at the original position. The proximal dilated ureter became the neo-orifice with a double J stent in the ureter (Figure 1F).

#### Pneumovesicoscopic Cohen Reimplantation

In pneumovesicoscopic Cohen reimplantation, the body position and establishment of the pneumovesicum in the bladder are similar to those employed in pneumovesicoscopic modified ureteral orthotopic reimplantation. Subsequently, a segment of a 3F ureteral stent was inserted 4–6 cm into the ureter. The following procedure was performed in a manner similar to the Cohen reimplantation procedure (7). A double J stent was inserted into the ureter through the tunnel at the end of the surgery.

### Follow-Up Procedure

**Figure 2:**
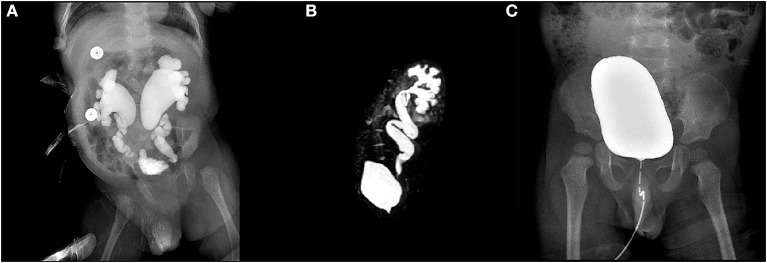
Voiding cystourethrography (VCUG) demonstrated bilateral reflux in one patient with grade V vesicoureteral reflux (VUR) **(A)**. Magnetic resonance urography demonstrated hydroureteronephrosis in one patient with primary obstructive megaureter (POM) **(B)**. VCUG did not demonstrate reflux 3 months after stent removal **(C)**.

Postoperative anti-infective therapy consisted of penicillins or cephalosporin for 48 h. Postoperative analgesia was not used in any of the patients. The ureteral stent was removed at 1 month post-surgery. Cystoscopy was performed while removing the stent to evaluate the new ureteral orifice, and VCUG was performed after stent removal. All patients underwent repeat ultrasound and VCUG (Figure 2C) at 3 months, 6 months, and 1 year after stent removal to monitor for obstruction or VUR. The diameter of the ureter was measured after voiding to determine the degree of dilatation.

## Statistical Analysis

Statistical analysis was performed using the Student's *t*-test for continuous variables and chi-squared test for categorical variables with the SPSS 13.0 software. A value *p* < 0.05 denoted statistical significance.

## Results

Surgical details and outcomes are summarized in Table 2. The operative time was significantly shorter in the OR group (OR: 86.86 ± 12.37 and 108.18 ± 15.70 min vs. CR: 95.14 ± 8.58 and 124.29 ± 13.97 min, for unilateral and bilateral cases, respectively; *p* < 0.05). There was no significant difference in postoperative hospital stay (OR: 5.02 ± 0.88 vs. CR: 5.07 ± 0.99 days; *p* > 0.05) and volume of blood loss (OR: 3.67 ± 0.90 vs. CR: 3.84 ± 0.81 ml; *p* > 0.05). Conversion to open surgery occurred in one case (3-month-old infant) in the CR group due to the limited bladder volume. Of note, there was no conversion in the OR group.

**Table 2:** Comparison of operative details and outcomes between modified ureteral orthotopic reimplantation (OR) and Cohen reimplantation (CR).

**Operative details and outcomes**	**OR (*n* = 46)**	**CR (*n* = 43)**	*p*-value^*^
Operation time (mean ± SD, min) Unilateral	86.86 ± 12.37	95.14 ± 8.58	<0.01
Bilateral	108.18 ± 15.70	124.29 ± 13.97	0.04
Volume of blood loss (mean ± SD, ml)	3.67 ± 0.90	3.84 ± 0.81	0.37
Postoperative hospital stay (mean ± SD, days)	5.02 ± 0.88	5.07 ± 0.99	0.81
Follow-up period (mean ± SD, months)	23.17 ± 10.14	23.37 ± 12.85	0.94
Improved hydroureteronephrosis (%)	46 (100%)	43 (100%)	
Preoperative ureteral diameter (mean ± SD, cm)	1.37 ± 0.41	1.35 ± 0.35	0.69
Postoperative ureteral diameter (mean ± SD, cm)	0.45 ± 0.20	0.47 ± 0.19	0.92
Preoperative renal pelvis AP diameter (mean ± SD, cm)	1.99 ± 0.34	1.98 ± 0.30	0.82
Postoperative renal pelvis AP diameter (mean ± SD, cm)	0.92 ± 0.34	0.90 ± 0.31	0.72
Reflux resolution for patients with VUR, *n* (%)^**^	8 (100%)	9 (100%)	
Re-operation	0	0	
Conversion to open surgery	0	1	0.28

* *p-values calculated using the chi-squared test for categorical variables and Student's t-test for continuous variables*.

** *Reflux resolution in patients with VUR in the two groups; there were eight and nine patients withVUR in the OR group and CR group, respectively. SD, standard deviation; VUR, vesicoureteral reflux; AP, antero-posterior*.

According to the Clavien–Dindo classification (8), postoperative complications include the following: Grade 1: febrile UTI, incision infection, and urinary retention. Two patients in each group experienced postoperative febrile UTI, while the stent was placed in the bladder. All infections were controlled using antibiotics according to the results of urine culture drug sensitivity test and resolved after stent removal without the requirement for long-term administration of oral antibiotics. There was no occurrence of incision infection and urinary retention. Grade 2: none. Grade 3: VUR, ureteral obstruction, bladder leak, and ureteral injury. Grades 4–5: none. Grades 2–5 postoperative complications did not occur.

**Figure 3:**
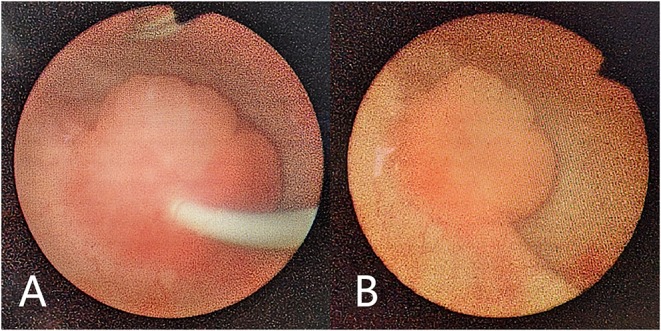
Cystoscopy performed while removing the ureteral stent. The protruding portion of the ureter in the bladder appeared nipple shaped with a ureteral stent in the ureter **(A)**. The protruding portion of the ureter in the bladder appeared nipple shaped **(B)**.

There was no significant difference in follow-up time (OR: 23.17 ± 10.14 vs. CR: 23.37 ± 12.85 months; *p* > 0.05). In all patients treated for VUR in both groups, the condition was completely resolved after removal of the ureteral stent. Two patients in the OR group and one patient in the CR group presented Grades I–II VUR after removal of the ureteral stent, as shown on VCUG. All three cases exhibited complete reflux resolution on repeated VCUG 3 months after stent removal. At the same time point, ultrasound examination revealed that the ureteral diameter (OR: from 1.37 ± 0.41 to 0.45 ± 0.20 vs. CR: from 1.35 ± 0.35 to 0.47 ± 0.19 cm; *p* > 0.05) and renal pelvis antero-posterior diameter (OR: from 1.99 ± 0.34 to 0.92 ± 0.34 vs. CR: from 1.98 ± 0.30 to 0.90 ± 0.31 cm; *p* > 0.05) were significantly decreased in all patients. There was no requirement for re-operation or cases of ureteral obstruction after removal of the ureteral stent in either of the groups. At the time of ureteral stent removal, we found that the protruding portion of the ureter in the bladder appeared nipple-shaped under cystoscopic vision (Figures 3A,B).

## Discussion

Over the last few decades, the management and treatment of VUR or POM in children have been drastically evolving. The first-line treatment for VUR is endoscopic subureteral injection, although it involves repeated injections (9). Moreover, the effectiveness of the endoscopic treatment technique used in cases with very high-grade reflux (grades IV–V) remains controversial (10). Surgery is the first choice for the treatment of very high-grade reflux and persistent POM; conventional management through surgery involves reimplantation of the ureter after excision of the distal ureteric segment (6, 11). The Cohen technique is widely applied as one of the most reliable procedures associated with excellent outcomes (12–14). McCool et al. reported a 96% rate of reflux resolution using the Cohen technique (15). Nevertheless, the major limitation of the Cohen technique is that it impedes future endoscopic retrograde access to the upper urinary tract of the child (1). This is attributed to the relocation of the ureteral orifice to the opposite side of the trigone. The Glenn–Anderson and Politano–Leadbetter techniques are intravesical procedures in which the normal anatomic configuration is not significantly altered (16, 17). However, it is difficult to obtain a sufficient tunnel length in cases with a small-capacity bladder or severely dilated ureter. Various techniques have been proposed to overcome this challenge in cases with a small-capacity bladder, namely, Kalcinsky plication, Starr plication, psoas hitch, and Hendren's excisional tapering (11). Although these techniques have yielded excellent results, a stiff segment may theoretically develop at a tapered distal ureter in a long tunnel (18). There is also a potential risk of bowel injury associated with the Politano–Leadbetter technique, which may occur during transvesical ureterolysis and reimplantation of the distal ureter (19). The Lich–Gregoir technique is also an effective procedure for extravesical ureteral reimplantation, which preserves the normal alignment of the ureter. The main disadvantage of the Lich–Gregoir technique is the risk of postoperative urinary retention (20–22). Neurovascular injury may be the cause of voiding dysfunction (23). Although this complication can be reversed, it has limited the application of this technique to bilateral cases.

We used the simpler procedure of ureteral reimplantation under pneumovesicum to overcome the disadvantages of the currently available techniques. In this intravesical approach, the new orifice was recreated in an orthotopic position without a cross-trigonal tunnel; hence, retrograde examination of the upper urinary tract would be feasible in the future. Compared with the classic Cohen reimplantation, this technique is easier and requires a shorter operation time, owing to the absence of tapering of the ureter or a cross-trigonal tunnel. Tatlisen and Ekmekcioglu (24) described a “nipple ureteroneocystostomy,” which is similar to our technique, and reported good outcomes. Compared with nipple ureteroneocystostomy, our technique is easier owing to the lack of ureter folding. However, the sample of this study was very small, and there was no granulation tissue on the new ureteric opening post-surgery following the nipple technique. In the present study, our technique showed equivalent effectiveness and safety compared with the Cohen technique. Two patients in the OR group presented Grades I–II VUR after stent removal and subsequently exhibited complete reflux resolution on repeated VCUG 3 months after removal of the ureteral stent. The occurrence of these complications may be attributed to the temporary open state of the terminal ureter after stent removal. The two cases occurred early in our experience with this technique.

Lyon et al. reported that the reflux rate appeared to be highly related to the shape, size, and configuration of the orifice (25). The mechanism of VUR prevention through our technique can be summarized in the following four points. First, the ureter was embedded between the bladder mucosa and bladder muscle to enable a tunnel of 1–1.5 cm in length and exposed into the bladder for 2–2.5 cm, making it possible for anti-reflux by bladder wall contraction and ureteral circular muscle. Second, the distal narrow segment of the ureter was dissected as the transitional segment remained at 3- to 4-mm ureteral diameter rather than at the dilated segment in the Cohen technique, which remained at 10-mm ureteral diameter. Third, the nipple-shaped orifice is relatively effective in preventing the occurrence of VUR (25). Finally, the new orifice was reimplanted at its original position. Our technique satisfies all the three elements (i.e., shape, size, and configuration) for the prevention of VUR, despite the relatively short tunnel length.

In this article, we reported a simple and feasible surgical technique under pneumovesicum as an alternative treatment for VUR or POM. Considering the limitation of the small sample size in our present study, future larger studies are warranted to define the role of this procedure in the treatment of pediatric patients.

## Data Availability Statement

All datasets generated for this study are included in the article/supplementary material.

## Ethics Statement

The studies involving human participants were reviewed and approved by Qilu hospital, Shandong University, Shandong, China. Written informed consent to participate in this study was provided by the participants' legal guardian/next of kin.

## Author Contributions

AL, XL, and JC contributed to the conception and design of the study. JC, QZ, PH, and DW retrospectively collected and analyzed the data. JC wrote the first draft of the manuscript. All authors reviewed the manuscript, and read and approved the submitted version.

### Conflict of Interest

The authors declare that the research was conducted in the absence of any commercial or financial relationships that could be construed as a potential conflict of interest.

## Abbreviations

POM: primary obstructive megaureter
VUR: vesicoureteral reflux
OR: orthotopic reimplantation
CR: Cohen reimplantation
UTI: urinary tract infection
VCUG: cystourethrography.
